# Sex trafficking and sexual exploitation in settings affected by armed conflicts in Africa, Asia and the Middle East: systematic review

**DOI:** 10.1186/s12914-016-0107-x

**Published:** 2016-12-28

**Authors:** Alys McAlpine, Mazeda Hossain, Cathy Zimmerman

**Affiliations:** Department of Global Health and Development, London School of Hygiene & Tropical Medicine, 15-17 Tavistock Place, Kings Cross, London, WC1H 9SH UK

**Keywords:** Sexual exploitation, Human trafficking, Sex trafficking, Child trafficking, Conflict, Forced marriage, Early marriage, Sex slavery, Forced conscription

## Abstract

**Background:**

Sex trafficking and sexual exploitation has been widely reported, especially in conflict-affected settings, which appear to increase women’s and children’s vulnerabilities to these extreme abuses.

**Methods:**

We conducted a systematic search of ten databases and extensive grey literature to gather evidence of sex trafficking and sexual exploitation in conflict-affected settings. International definitions of “sexual exploitation” and “sex trafficking” set the indicator parameters. We focused on sexual exploitation in forms of early or forced marriage, forced combatant sexual exploitation and sexual slavery. We extracted prevalence measures, health outcomes and sexual exploitation terminology definitions. The review adhered to PRISMA guidelines and includes quality appraisal.

**Results:**

The search identified 29 eligible papers with evidence of sex trafficking and sexual exploitation in armed conflict settings in twelve countries in Africa, Asia, and the Middle East. The evidence was limited and not generalizable, due to few prevalence estimates and inconsistent definitions of “sexual exploitation”. The prevalence estimates available indicate that females were more likely than males to be victims of sexual exploitation in conflict settings. In some settings, as many as one in four forced marriages took place before the girls reached 18 years old. Findings suggest that the vast majority of former female combatants were sexually exploited during the conflict. These studies provided various indicators of sexual exploitation compatible to the United Nation’s definition of sex trafficking, but only 2 studies identified the exploitation as trafficking. None of the studies solely aimed to measure the prevalence of sex trafficking or sexual exploitation. Similar descriptions of types of sexual exploitation and trafficking were found, but the inconsistent terminology or measurements inhibited a meta-analysis.

**Conclusions:**

Findings indicate there are various forms of human trafficking and sexual exploitation in conflict-affected settings, primarily occurring as early or forced marriage, forced combatant sexual exploitation, and sexual slavery. The studies highlight the extraordinary vulnerability of women and girls to these extreme abuses. Simultaneously, this review suggests the need to clarify terminology around sex trafficking in conflict to foster a more cohesive future evidence-base, and in particular, robust prevalence figures from conflict-affected and displaced populations.

## Background

Recent attention to sexual violence in conflict settings has begun to indicate how displacement, instability and the collapse of laws and basic services can increase the risk of sex trafficking and sexual exploitation, including: early or forced marriage, sexual exploitation of military combatants and sex slavery [1]. Global estimates suggest that roughly 36–62% of the 20.9 million people who are estimated to be in situations of forced labor or human trafficking are trafficked for sexual exploitation [2].

Definitional questions continue to plague the field of human trafficking, especially trafficking for sexual exploitation [3]. The most widely accepted definition of human trafficking is found in the United Nation’s (UN) Convention Against Transnational Organized Crime and its Protocol to Prevent, Suppress and Punish Trafficking in Persons, Especially Women and Children:

The recruitment, transportation, transfer, harbouring or receipt of persons by means of threat or use of force or other forms of coercion, of abduction, of fraud, of deception, of the abuse of power, or of a position of vulnerability or of the giving or receiving of payments or benefits to achieve the consent of a person having control over another person, for the purpose of exploitation. Exploitation shall include, at minimum, the exploitation of the prostitution of others or other forms of sexual exploitation, forced labour or services, slavery or practices similar to slavery, servitude or the removal of organs [4].

While controversies persist around the application of the term ‘trafficking’, most experts agree that the fundamental feature that defines human trafficking is the act of “exploitation”—generally in extreme forms (forced labor, slavery) [5, 6].

Vulnerability to these extreme forms of exploitation, particularly sexual exploitation, are driven or facilitated by both individual factors (e.g., household income, social status), and structural factors, especially the breakdown of legal structures, social networks and livelihood options in settings affected by conflict and displacement [7]. Human trafficking, particularly involving forced sex, has been associated with serious physical, sexual and psychological abuses [8]. In 2016, there are an estimated 40 active conflicts, 65.3 million forcibly displaced people and 21.3 million refugees worldwide [9, 10]. Crisis conditions generally exacerbate existing hardships and create opportunities for traffickers to profit from the exploitation of desperation. In the case of sex trafficking, gender inequities and the disproportionate disadvantages faced by women and girls increase their risk of coercion, exploitation and abuse. While aid workers in humanitarian crisis settings are increasingly aware of the rise in human trafficking among the populations they assist, there has been little guidance on how to help prevent trafficking of these particularly at-risk populations or how to identify or provide assistance to those who have fallen prey to traffickers. For example, a 2014 review of guidance documents addressing human trafficking in conflict-affected settings identified fourteen documents offering some form of information for staff working in emergency settings, such as refugee and internally displaced persons (IDP) camps, yet, none focused specifically on trafficking in conflict *and* also offered operational guidance (e.g., general guidance on gender-based violence with information on trafficking; legislative summaries on trafficking in situations of conflict) (A. Whiting, A. Stepnitz, J. Freccero, and C. Zimmerman: The Case for Operational Guidance to Prevent and Respond to Human Trafficking in Conflict-Affected Settings, unpublished). The International Organization for Migration (IOM) recently released a report on trafficking and exploitation in times of humanitarian crisis and it is the first report to make operational recommendations for a response strategy during the various stages of a crisis (before, during and after) [11]. Given the poor state of evidence about trafficking of individuals affected by war, it is not surprising that there is little guidance on intervention approaches for prevention or assistance.

The original aim of this review was to collect and synthesize prevalence data. However, as will be discussed, in reviewing the literature it is evident that the current definitions and methods used to measure sex trafficking and sexual exploitation are too heterogeneous to synthesize in a meta-analysis. Instead, this review aims to inform future policy, research and programming responses to sexual exploitation and sex trafficking in conflict-affected settings by reviewing the types of sex trafficking and sexual exploitation measured in conflict-affected settings and present the varied terminology use, and discuss the dynamics of these different violence exposures through reviewing prevalence indicators and health outcomes.

## Methods

### Defining sexual exploitation and sex trafficking

The first task of this review was to address some of the terminological challenges because of the definitional complexities of human trafficking, especially those surrounding ‘sex trafficking’. The authors drew on the UN Convention Against Transnational Organized Crime and the associated Protocol to Prevent, Suppress and Punish Trafficking in Persons, Especially Women and Children’s definition of human trafficking and the UN Secretary-General’s definition of ‘sexual exploitation’ to set terminological parameters for the search. Specifically, sexual exploitation has been delineated based on the UN Secretary-General’s Bulletin on protection from sexual exploitation and abuse:

The term “sexual exploitation” means any actual or attempted abuse of a position of vulnerability, differential power, or trust, for sexual purposes, including, but not limited to, profiting monetarily, socially or politically from the sexual exploitation of another. Similarly, the term “sexual abuse” means the actual or threatened physical intrusion of a sexual nature, whether by force or under unequal or coercive conditions [12].

Paired with the UN definition of human trafficking, this definition of sexual exploitation provided the parameters for this evidence review and search focused specifically on forms of sexual exploitation prominent in conflict-affected populations that also employed the same acts and means used to define cases of trafficking [4]. Sexually exploited combatants have been included because of the coercive and often unavoidable nature of recruitment and conscription. Early and forced marriage have likewise been included as the absence of consent, informal detainment, and implied sexual nature of the relationship create grounds for inclusion [13]. The UN definition states that trafficking includes “practices similar to slavery”, which indicates the inclusion of these particular vulnerable groups and all mentions of sexual slavery or servitude with forced sex [4]. We employ these definitions for this review, while acknowledging that terminology has and will continue to evolve for various forms of gender-based violence, including sex trafficking and sexual exploitation.

### Search strategy and selection criteria

This review adheres to the Preferred Reporting for Systematic Reviews and Meta-Analysis (PRISMA) guidelines [14]. Ten databases were searched; Embase, London School of Hygiene and Tropical Medicine (LSHTM), Journals, Medline, New York Academy of Medicine (NYAM), Popline, PsychInfo, ReliefWeb, Studies on Women and Gender Abstracts (SWGA), Web of Science, and World Health Organization (WHO) Reproductive Health Library (RHL). To capture research published outside of the peer-reviews literature, we also used a grey-literature search approach and bibliographic reference searching. Articles and documents published between January 1, 2000 and August 1, 2014 were included in the review.

Inclusion criteria were as follows: studies conducted in any armed conflict settings in Africa, Asia, and/or the Middle East that had on-going conflicts as of January 1, 2000 or commenced after this date; studies with empirical evidence of any form of relevant sexual exploitation (as previously described) or sex trafficking reported separately from other indicators; and studies with clearly reported methodology regardless of study design or type of data. Victims of every gender, age, sexual orientation, and nationality were included from all types of conflict settings. Studies reported in languages other than English were excluded due to the capacity of the research team. Any study or report that reported on violence solely perpetrated by humanitarian workers and/or peacekeepers was excluded as this specific type of perpetrator inflicted violence is covered in depth by other reviews and agency reports that were deemed to be outside the scope and aim of this review [15, 16].

This review used an advanced search of the two concepts of interest, sex trafficking and armed conflict, based on expert consultation and an initial literature review using key terms. The following search strategy was used in PubMed:

Key term 1: [(sex* adj3 traffick*) or sex* trade or (sex* adj3 exploit*) or (sex* adj3 abduct*) or (sex* adj3 slave*) or forced prostitute* or child* prostitute* or arranged marriage or early marriage or forced marriage or child* bride or child* soldier or kidnap* or brothel]

AND

Key term 2: [armed conflict* or war* or combat* or refugee or (complex adj3 emergency) or terroris* or military* or (rebel adj3 group) or genocide or army or soldier]

**Fig. 1:**
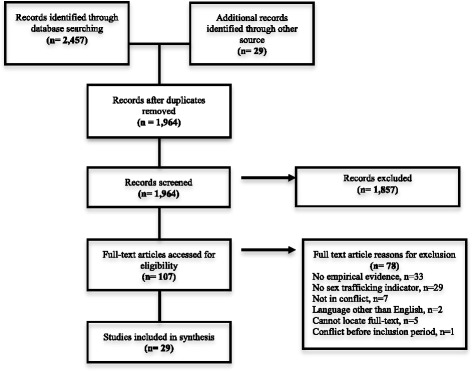
PRISMA flow diagram of review search [14]

A simpler two-concept search [(sex exploitation OR sex trafficking) AND (conflict OR war)] was employed for any database that did not allow advanced searches, for both peer-reviewed and grey literature sources. Online specialist libraries were screened in most cases for the 40+ grey literature site searches. The study selection process is presented in Fig. 1. The total yield was 2,486, but after removing for duplication and applying the exclusion criteria only 29 studies met the inclusion criteria.

### Quality assessment and data extraction

The quality of each study was appraised using the Critical Appraisal Skills Program (CASP) to assess the strength of the evidence [17]. Each study received a final percentage score based on the number of CASP quality criteria that the study met in its described methods. Medium to high quality studies were those studies that received a CASP quality score of 75% or higher.

### Bias

Publication bias was minimised by searching bibliographic databases that included grey literature and hand searching for grey literature. Possible bias was introduced by excluding studies in languages other than English. However, ultimately only two out of the original 2,486 yield were excluded for this reason. If multiple articles presented the same study only the more descriptive of the two articles was included.

### Data extraction

Studies were classified by the type of data (quantitative or qualitative) relevant to the indicators of interest. Study design information extracted included: study design; sampling method; main objectives; data collection tools used; country; study setting; sample population; and the status of the conflict at the time the research was conducted. The primary data of interest was extracted for all studies: sex trafficking indicators; prevalence estimates; and indicator definitions. Secondary data of interest was also collected to enhance understanding of the context. For quantitative studies we noted the proportion of perpetrators that were military combatants and for qualitative studies any of doesn’t make sense as is the thematic findings on risk factors, abduction, perpetrators, and adverse health outcomes were extracted. Thematic findings from qualitative studies were summarised.

The sex trafficking and sexual exploitation data were classified into three categories; 1) early or forced marriage, 2) sexual exploitation of combatants, and 3) sex slavery. There was some overlap in definitions so data was classified in a mutually exclusive manner. Early or forced marriage was seen as the most specific and exclusive category where sex slavery had the broadest inclusivity for any indicator not pertaining to the other two categories. Terminology indicating both the trafficking act and sexually exploitive purposes were considered essential. For example, measuring levels of sexual violence in an IDP camp alone did not meet the inclusion criteria, because this broad measurement would include incidents of intimate partner violence not included as a form of trafficking in this review. Similarly, studies measuring single incidents of rape of non-partner violence unrelated to larger acts of coercion or abduction (or any other indicators of trafficking *acts* and *means*) were excluded. However, an act of *abduction* and rape in an IDP camp was included in the review.

### Analysis

Given the heterogeneity of definitions and methodologies, a meta-analysis was not possible. Therefore this review opted to categorize the sexual exploitation and sex trafficking indicators and present the research findings by type.

## Results

**Fig. 2:**
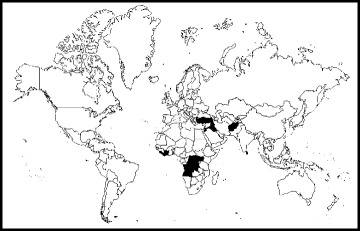
Countries included in review evidence [18] Africa: Angola, Cote d’Ivoire, Democratic Republic of the Congo, Liberia, Sierra Leone, Uganda, Middle East: Iraq, Jordan, Lebanon, Turkey, Asia: Afghanistan, Sri Lanka

Twenty-nine studies were identified for inclusion, fourteen quantitative and fifteen qualitative. The geographical locations of the studies are represented in Fig. 2, which include twelve countries across Africa, Asia and the Middle East, with half of the study settings in Africa [18].

### Quality

CASP quality scores ranged from 35–97% with an average score of 72%. Quality was frequently detracted due to poorly defined exposures and outcomes, lack of statistical precision in the case of quantitative studies, unclear sampling design in the case of qualitative studies, and for all the studies there was an issue of generalizability due to unrepresentative samples. Out of the 29 studies only two were of high quality and both were quantitative studies. One was a population based survey measuring war trauma in Liberia through mental health surveys. The second was a university-based survey that gathered evidence of war-affected youths’ experiences of forced marriage and sex slavery during the conflict in Uganda.

### Findings

Tables 1, 2 and 3 highlight the definitions and descriptors used across the twenty-nine studies. The definitions are not significantly different, but there is a notable absence of (and urgent need for) more cohesive definitions of the different forms of sexual exploitation present in conflicts to enable comparison across studies and regions. The table is organized into the three categories of exploitation employed by this review: 1) early or forced marriage; 2) sexual exploitation of combatants; and 3) sex slavery. The key terms indicated in bold along with the description or working definition that was provided by the study were extracted directly from the text of the study. For studies in which ‘abduction’ (ie. kidnapping, slavery, servitude, etc.) was identified in association with some form of sexual violence, both of these terms were extracted and included in Tables 1, 2 and 3.

**Table 1:** Early or forced marriage terminology as defined by the studies in this review

Author	Early or forced marriage [page number]
Amaireh [19]	early marriage- any marriage where one or both persons are under 18 years and classified as children under international law [pg 14] forced marriage- any marriage where one or both parties have not given their consent, including early marriage [pg 14] temporary marriage- any marriage that is contractually time-limited and can last as little as a few days or even hours up to several years, and is sometimes accompanied by a one-time dowry payment which is often paid to the girl’s male guardian, these marriages can act as a form of sanctioned casual sex and even prostitution [pg 33]
Amowitz, [20]	forced marriage to combatants- *no definition* [pg 518]
Bartels [26]	child marriage- a formal marriage or informal union before age 18, also referred to as early marriage [pg 38–39]
Brosnan [27]	early and/or forced marriage- *no definition*, but recognized as an act against female victims only [pg 2]
Carlson [28]	forced marriage- coercive relationships without valid consent of the female and her family with the traditional characteristics of shared domicile, bearing of children, domestic responsibilities, exclusivity and sex [pg 14], but these marriages do not have any legal standard [pg 8]. Forced marriage includes constituent acts that are codified crimes in international customary and human rights law. These crimes include rape, sexual slavery, enforced pregnancy, forced labor, enslavement and torture. [pg 14–15]
Duroch, [21]	forced wife- *no definition* [pg 6]
Gottschalk, [29]	early marriage- marriage under the age of 18 [pg 3]
Higonnet [30]	“wives” of rebels- abducted women forced to marry rebels [pg 41–42]
Johnson [38]	forced marriage- *no definition* [pg 557]
Kaya [47]	forced marriage- *no definition*, but includes child marriage [pg 44]
Kinyanda [23]	forced marriage- *no definition* [pg 4]
Kippenberg [31]	force marriage- *no definition*, but gives the example of individuals made to marry a soldier under threat of death [pg 30]
Kottegoda [24]	early marriage- marriage before the age of 18 [pg 78]
Okello [25]	forced to marry- *no definition*, but recognized as an act against female victims only [pg 227]
Save the children [32]	early marriage- marriage before the age of 18, also referred to as child marriage [pg 3]
Schlecht [33]	child marriage- marriage before 18 years of age [pg 1], also called early marriage forced marriage- *no definition* [pg 235]
Stavrou	war husbands- *no definition* [pg 20]
Vindevogel [40]	rebel wife- *no definition* [pg 555]
Weber [35]	“wives” of Lord’s Resistance Army (LRA) commanders- forced sexual slavery, subjected to rape, forced pregnancy, and the risk of sexually transmitted diseases, including HIV/AIDS [pg 5]

**Table 2:** Sexual exploitation of combatants terminology as defined by the studies in this review

Author	Sexual exploitation of combatants [page number]
Bayer [36]	former child soldiers*-* an individual under 18 years old that reported being (often violently) recruited by armed forces [pg 555] forced sexual contact- *no definition* [pg 558]
Betancourt [37]	child soldier- both boys and girls who are part of any kind of regular or irregular armed force or armed group in any capacity, including, but not limited to: cooks, porters, messengers and those recruited for sexual purposes and for forced marriage [pg 21] sexual abuse or sexual assault- *no definition*, but rape and forced marriage mentioned as two examples [pg 22]
Betancourt [52]	child soldier- *no definition* [pg 1] sexual violence- *no definition* [pg 1] rape- *no definition* [pg 1]
Carlson [28]	LRA combatant- LRA rebels fighting the government of Uganda (the study was looking at LRA perpetrated abductions) [pg 4] abduction- *no definition*, absence of dowry is used synonymously with abduction [pg 52] sexual violence- *no definition* given but examples used are rape, sexual based crimes, gender based crimes, forced impregnation [pg 6, 15]
CSUCS [53]	child soldier- A child associated with an armed force or armed group refers to any person below 18 years of age who is, or who has been, recruited or used by an armed force or armed group in any capacity, including but not limited to children, boys and girls, used as fighters, cooks, porters, spies or for sexual purposes. It does not only refer to a child who is taking, or has taken, a direct part in hostilities. (UNCEF 2007) [pg Title page] sexual violence- *no definition*, but includes sexual abuse and rape [pg 28] LRA sex slaves- *no definition* [pg 17]
Denov [42]	child soldier- any person below 18 years of age who is or who has been recruited or used by an armed force or armed group in any capacity, including but not limited to children, boys and girls, used as fighters, cooks, porters, messengers, spies or for sexual purposes. It does not only refer to a child who is taking or has taken a direct part in hostilities [pg 805] sexual violence- sexual labor, sexual assault, systematic raping, sexual exploitation [pg 794]
Denov [43]	child soldier- children engaged in the conflict, child combatants [pg 74] sexual violence- gang rape, individual rape and/or rape with objects, also referred to as sexual slavery, sexual harassment, sexual victimization [pg 77]
Higonnet [30]	female child soldiers- girls formerly associated with the armed conflict as soldiers, cooks, porters, sex slaves, or in some combination of these roles [pg 37] sexual violence- sexually exploitive acts including individual and gang rape, sexual slavery, forced incest, and egregious sexual assault [pg 3] and defined as a form of GBV along with sexual abuse, domestic violence, emotional and psychological abuse, trafficking, forced prostitution, sexual exploitation, sexual harassment, and harmful traditional practices (e.g. female genital mutilation, forced marriage, or widow cleansing) [pg 131]
Johnson [38]	combatant- any person who reported being part of any kind of regular or irregular armed force in any capacity, including but not limited to participation in combat, laying mines or explosives, serving as a cook or domestic laborer, decoy, courier, guide, guard, porter, or spy, trained or drilled as a combatant, or serving as a sexual servant/slave [pg 680] sexual servitude- individual forced to be a sexual servant or sexual slave to a government or nongovernment military or militia group at any point in their life [pg 680] sexual violence- any violence, physical or psychological, carried out through sexual means or by targeting sexuality and included rape and attempted rape, molestation, forced undressing, forced intercourse, or other sexual acts [pg 680] sexual slavery, being forced to undress or being stripped of clothing, forced marriage, and insertion of foreign objects into the genital opening or anus, forcing 2 individuals to perform sexual acts on one another or harm one another in a sexual manner, or mutilating a person’s genitals [pg 680]
Klasen [39]	child soldier- any person under 18 years of age associated with an armed force or armed group in any capacity ranging from combatants to cooks [pg 1098] sexual violence- *no definition*, but includes acts of sexual insults, sexual touching, rape (also referred to as sexual abuse and sexual trauma) [pg 1102]
Stavrou [34]	child soldiers- children under the age of 18 serving in the armed forces, also called underage soldiers [pg 12] sex labor- *no definition*, but the primary example used was rape [pg 45]
Vindevogel [40]	child soldiers- any person under 18 years of age who is part of any kind of regular or irregular armed force or armed group in any capacity, including but not limited to cooks, porters, messengers, and anyone accompanying such groups, other than family members, including girls recruited for sexual purposes and for forced marriage [pg 553] sexual abuse- *no definition* [pg 556]
Weber [35]	abductees- individuals taken by the military and used as soldiers, porters, laborers, and in the case of girls, as sexual slaves [pg 11]

**Table 3:** Sex slavery terminology as defined by the studies in this review

Author	Sex slavery [page number]
Amowitz [20]	sex slavery- *no definition* [pg 518] sexual violence- acts including rape and other forms of sexual assault, such as molestation, sexual slavery, being forced to undress or being stripped of clothing, forced marriage, and insertion of foreign objects into the genital openings or anus, gang rape which is defined as rape by 2 or more individuals [pg 516]
Bartels [45]	sexual assault- acts included genital mutilation, instrumentation with foreign objects, forced rape between victims, rape in the presence of family members, anal penetration, forced oral sex, sexual harassment, forced to undress, forced rape between victims and insertion of foreign objections into the vagina or anus [pg 2,16] sexual slavery- being held captive for the purpose of sexual violence for more than 24 h [pg 9]
Duroch [21]	detention or kidnapping- act of confinement taking place before or after an attack [pg 2,5] sexual violence- predominately refers to rape but includes any act of a sexual nature which is committed on a person under circumstances which are coercive, this includes forced vaginal and anal intercourse, oral sex, penetration with foreign objects and forced undressing in public, and gang rape, which refers to sexual assault by two or more aggressors [pg 4]
Higonnet [30]	sex slaves- the status or condition of a person over whom any or all of the powers attaching to the right of ownership are exercised, including sexual access through rape or other forms of sexual violence, and includes most forms of forced prostitution [pg 41]
Johnson [22]	sexual violence- any physical or psychological violence carried out through sexual means or by targeting sexuality and included rape and attempted rape, molestation, sexual slavery, being forced to undress or being stripped of clothing, forced marriage, and insertion of foreign objects into the genital opening or anus, forcing individuals to perform sexual acts on one another or harm one another in a sexual manner, or mutilating a person’s genitals and gang rape which was defined as rape by 2 or more individuals [pg 555] sexual servitude- individual forced to be a sexual servant or sexual slave to a government or nongovernment military or militia group at any point in their life [pg 555]
Kaya [47]	sexual servitude- assault where the victim is physically molested or raped by the traffickers [pg 44], listed as a form of trafficking [pg 9,14]
Kinyanda [23]	abduction with sex- *no definition* [pg 4]
Kippenberg [31]	sexual slavery- including enforced prostitution and forced pregnancy, the victim is detained for an extended period [pg 17, 29]
Nelson [46]	sexual violence- any unwanted physical contact of a sexual nature, including gang rape, which was defined as sexual violence committed by two or more assailants [pg 214] sexual slavery- being held captive for the purpose of sexual violence for >24 h [pg 214–215]
Okello [25]	war abduction- any child who had been forcefully taken away by armed forces [pg 226] sexual torture- *no definition* [pg 227]

The data in Tables 4 and 5 are presented in the same three categories, type of sexual exploitation or trafficking, described in Tables 1, 2 and 3. Within this classification, evidence is presented for both qualitative and quantitative studies. All 29 studies included an indicator that this review defines as sex trafficking in conflict or post-conflict, although no study’s primary objective was solely to measure this prevalence. The studies used various methods of data collection such as medical records, counselling records, surveys, in-depth interviews and focus group discussions. One of the most significant findings of this review is the varied definition of key terms: early marriage, forced marriage, child soldier, sexual exploitation, and sex slavery. Only two studies distinguished the indicators of relevance as “human or sex trafficking” and despite wide acceptance of the umbrella term “gender-based violence” for exposures such as the ones included in this review, only eleven of the twenty-nine studies defined the indicator as an act of gender-based violence (GBV).

**Table 4:** Findings from the quantitative studies (*n =* 14)

Author (Year)	Country	Setting	Study population	Average age in years	Type of sexual exploitation or trafficking	% of female sample victimized (95% CI)^a^ [n/total female]	% of male sample victimized (95% CI)^a^ [n/total male]	% total sample victimized (95% CI)^a^ [n/total]	% Perpetrator sample that were military personnel (95% CI)^a^ [n/total]	Quality score
Amaireh [19]	Jordan	Refugee camps	Syrian refugees living in Jordan	-	Early or forced marriage	51.3%	13.0%	33.2%	-	67%
Amowitz [20]	Sierra Leone	Internally displaced persons camps	Heads of households Avg. 34 years	34	Sex slavery	1.5% [15/991]	-	1.5% [15/991]	-	80%
					Early or forced marriage	1.0% [9/991]	-	1.0% [9/991]	100% [9/9]	
Bartels [45]	DRC	Panzi Hospital (specialty hospital for GBV victims)	Sexual violence survivors requesting services, Avg. 35 years	35	Sex slavery	12.0% [573/4778]	-	-	-	43%
Bayer [36]	DRC	Rehabilitation centers	Former child soldiers Avg. 15.3 years	15.3	Sexual exploitation of combatants	57.1% [16/28]	22.0% [31/141]	27.8% [47/169]	-	63%
Betancourt [37]	Sierra Leone, post-conflict regions	Conflict affected regions	Former child soldiers Avg. 16.5 years	16.5	Sexual exploitation of combatants	44.3% [35/79]	5.0% [9/194]	16.0% [44/273]	100%	67%
Duroch [21]	DRC	MSF sexual violence clinics	Survivors of sexual violence, Avg. 25.7 years	25.7	Sex slavery	16.6% [408/2462]	65.1% [67/103]	18.5% [475/2565]	-	53%
					Early or forced marriage	3.5% [86/2462]	0.0% [0/103]	4.0% [86/2152]		
Johnson [38]	Liberia	Conflict affected regions	1666 adults	-	Sexual exploitation of combatants	9.1% [80/880]	15.01% [118/786]	11.2% [198/1666]	86.1% [167/194]	93%
Johnson [22]	Eastern DRC	Conflict affected regions	Household heads who are 18 years+, Avg. 41 years	41	Early or forced marriage	3.4% (0.0–0.1) [6/202]	0.0% [0/88]	2.0% [6/290]	-	77%
					Sex slavery	21.1% [52/222]	19.6% [24/103]	23.0% [76/325]		
Kinyanda [23]	Uganda	Internally displaced persons camps	IDPs	-	Sex slavery	7.9% [45/573]	1.7% [4/240]	6.0% [49/813]	98.0% [49/50]	73%
					Early or forced marriage	4.4% [25/573]	1.7% [4/240]	3.6% [29/813]	86.2% [25/29]	
Klasen [39]	Uganda	Boarding schools for conflict affected youth	Former child soldiers	14.4	Sexual exploitation of combatants	29.4% [47/160]	22.4% [38/170]	25.8% [85/330]	100%	80%
Kottegoda [24]	Sri Lanka	Conflict affected regions	Women from various districts	-	Early or forced marriage	31.0%	-	-	-	87%
Nelson [46]	DRC	Panzi Hospital (specialty hospital for GBV victims)	Survivors of sexual violence	-	Paediatric sex slavery (<18 years)	17.2% [67/389]	-	-	100%	67%
					Adult sex slavery	12.4% [430/3458]	-	-	100%	
Okello, [25]	Uganda	Youth organizations and universities	War-affected adolescents, Ages 11–19 years	-	Early or forced marriage	-	-	11.8% [18/153]	100%	97%
					Sex slavery	-	-	11.1% (17/153)	100%	
Vindevogel [40]	Uganda	Former child soldier at rehabilitation centers	Former child soldiers	14	Sexual exploitation of combatants	56.0% [337/654]	0.0% [0/1,176]	18.4% (337/1,830)	-	63%

^**a**^The 95% confidence interval (95%CI) and/or percentage numerator and denominator [n/N] is included in the table if it was provided by the study. If not listed it means the study did not report these figures

**Table 5:** Findings from the qualitative studies (*n =* 15)

Author (Year)	Country	Setting	Study population	Type of sexual exploitation or trafficking	Thematic findings on risk factors	Thematic findings on abduction	Thematic findings on perpetrators	Thematic findings on adverse consequences/outcomes	Quality score
Bartels [26]	Lebanon	Refugee camps	Syrian refugees, Ages 18+ years	Early or forced marriage	informal tents, economic insecurity, need for employment	Families need to be made more aware of the risk of abduction.	Perpetrators were host country men, employers, aid workers, and family members.	increased social/physical abuse, maternal mortality, human trafficking, commercial sexual exploitation, sexually transmitted diseases (STDs), HIV	70%
Betancourt [37]	Sierra Leone	Conflict affected regions	Former child soldiers Age 10–17 caregivers, Former child soldiers’ relatives, key informants	Sexual exploitation of combatants	-	A high majority of the sample youth reported joining the Revolutionary united Front (RUF) by force/abduction.		stigma, decline in adaptive and pro-social behaviours, internalizing problems	70%
Brosnan [27]	Jordan, Turkey, Lebanon, Iraq	Refugee camps and urban displacement	Refugees, Government officials, IGO representatives, Non-governmental organization (NGO) staff	Early or forced marriage	displacement, economic insecurity, walking to school, rape	Families used early marriage as a way to safeguard their daughters honour.	Perpetrators were family members, host country citizens and armed forces personnel.	shame, stigma, anxiety, trauma, interrupted education, repeated rape	45%
Carlson [28]	Uganda	Internally displaced persons camps	Formerly abducted women/girls Additional use of key informants to target group	Early or forced marriage, Sexual exploitation of combatants	customary practices, economic hardship, puberty, living in an internally displaced persons camp, youth	Abduction was carried out by the Lord’s Resistance Army personnel and the field commanders had priority to forced marriages.	Often field commanders were the greatest culprits with multiple wives.	pregnancy, physical harm, mental harm, separation from family, death	70%
CSUCS [53]	DRC	Conflict affected regions, North and South Kivu	Military officials, government officials, NGO workers, child protection workers, community members, relatives	Sexual exploitation of combatants	war, absence of parents vulnerable children, armed groups, displacement, legal protection	-	The perpetrators were members of the Forces Armées de la République Démocratique du Congo (FARDC).	injury, death	50%
Denov [42]	Sierra Leone	Conflict affected regions	RUF former child soldiers Age 14–21 (all were 18 or under at time of exposure)	Sexual exploitation of combatants	war, widespread impoverishment, the breakdown of human security, and the gradual atomization of families and communities	Abductions often lasted between 2–18 months.	Victims were abducted by RUF soldiers.	physical, psychological, and social effects, community rejection, education dropout,	60%
Denov [43]	Sierra Leone	All regions	Former child soldiers	Sexual exploitation of combatants	war, unprotected children, fragmented political economy, disempowered women	All the participants had been abducted by the RUF under circumstances of extreme coercion, violence, and fear.	The RUF were most often responsible for abductions.	depression, violent injuries, pregnancy, stabbing, vomiting	60%
Gottschalk [29]	Uganda	Refugee camps	Refugees	Early or forced marriage	financial constraints, displacement, absence of parents, reduced livelihood options, war, untrained police, limited protective services, extramarital sexual relationships, physical insecurity	-	Often it is parents or guardians arranging the early marriages.	physical injury, social stigmas, rejection from family, school drop out	65%
Higonnet [30]	Cote d’Ivoire	Conflict affected regions	Survivors and witnesses of sexual violence	Early or forced marriage, Sexual exploitation of combatants, Sex slavery	low status of women and girls, conflict, low social status, economic disadvantage, traveling employment, political leaders wives and family members, displacement	-	Often girls were abducted by combatants and when they resisted abduction they were physically punished.	death, unwanted pregnancy, STIs, anxiety, shame, anger, depression, and fear	65%
Kaya [47]	Afghanistan	All regions	victims of trafficking or kidnapping, smuggled migrants, key informants	Sex slavery	protracted conflict, insecurity, limited access, instability, poverty, lack of trafficking awareness, loss of livelihood, high proportion of widows/orphans/people with disabilities, criminal networks, multiple neighbouring countries	A majority of trafficking victims are abducted under the lure of a better life or positive outcome and the remaining are kidnapped by force.	Many traffickers are involved in complex criminal networks. Often an individual's own family will sell them.	stigmatization, psychological harm, physical distress, pregnancy, loss of education	80%
Kippenberg [31]	DRC	Conflict affected regions	victims of rape, relatives, witnesses, community members, military combatants	Early or forced marriage, Sex slavery	conflict insecurities, insufficient pay for soldiers	-	The sample reported that the sexual exploitation was predominantly committed by the 14th brigade of the FARDC.	injury, death	70%
Save the Children [32]	Jordan	Refugee camps	refugees	Early or forced marriage	poverty, insecurity, fear of violence, conflict, youth	Many Syrian refugee families arranged the daughters’ weddings to Jordanian men.	-	poverty, loss of education, separation from family and friends, limited access to reproductive health, physical harm, mental and emotional strain, domestic violence, premature pregnancy	35%
Schlecht [33]	Uganda	Refugee camps	Ugandan and Congolese refugees	Early or forced marriage, Sexual exploitation of combatants	conflict, poverty, divided family, school dropout, early relationships, loss of livelihood	Families often planned early marriages and bride prices. During conflict there is militia-perpetrated abduction, forced marriage, and sex slavery.	-	poor health outcomes, poor social outcomes, early sexual debut, high risk pregnancy, limitations in negotiating condom use, STDs, school dropout, limited economic opportunity	70%
Stavrou [34]	Angola	Conflict affected regions	Formerly abducted girl soldiers Avg. 21 years	Early or forced marriage	combat zones, presence of soldiers, youth, displacement	-	The perpetrators were most often military personnel.	STDs, pregnancy, exhaustion, malnutrition, TB, abuse, death	60%
Weber [35]	Uganda	Conflict affected regions	victims of military violence, relatives of victims, and former LRA abductees	Early or forced marriage, Sexual exploitation of combatants	conflict, youth, displacement, travel	-	The perpetrators were most often military personnel.	unwanted pregnancy, STDs, injury, death	50%

### Early or forced marriage

Over half of the total number of studies collected data on incidents of early or forced marriage in conflict-affected populations [19–35]. Most of the studies were population-based surveys within war-affected regions, refugee or IDP camps.

Seven of the seventeen studies that showed evidence of early or forced marriage collected quantitative data [21, 24–35]. In four of the studies, female and male early or forced marriages were reported separately and all of these studies indicated higher prevalence for females (3–51%) compared to males (0–13%) [20, 22, 23]. In Uganda, a comparison of abducted and non-abducted war-affected adolescents showed that the abducted cohorts had 19.59 (CI 2.53–151.7) greater odds of being forced to marry than the non-abducted [25]. Studies identified an exposure to risk of sexual exploitation for all individuals in armed conflicts, male or female and abducted or not, but based on the indicated studies a greater risk for female and abducted groups [19–35].

When identifying the individual responsible for the forced marriage, both the qualitative and quantitative research found that a majority of perpetrators were affiliated with either the government or opposing military groups [20, 25, 27, 28, 30, 31, 34, 35].

In several first-hand accounts of early or forced marriage many conflict-affected families described early marriage as unavoidable in light of the household financial strains and risk of sexual violence faced during war. For example, a father of a Syrian child bride in a refugee camp explained that he “didn’t feel that [they] had any stability living in a place like this, with an enormous number of refugees. The instability left [him] with no choice but to make a decision…get her married off [32]”.

All ten of the qualitative studies that offer accounts of early or forced marriage indicate that the circumstances created by conflicts become risk factors for early or forced marriage [26–35]. The research suggests that early marriage is associated with certain factors that are present before conflict, such as cultural practices, while others are generated or exacerbated by conflict, such as sudden and extreme poverty [32]. In some of the studies, household representatives commented that early marriage was a means to cope with financial need by either receiving a payment for their daughter’s marriage or reducing the household’s expenses of having their daughter as a dependant [27, 32, 33]. Two studies reported that early marriage is seen as an alternative to finishing an education when girls’ transport to and from school and her attendance is perceived as too dangerous [29, 33]. Other studies described how conflict-related vulnerabilities, especially in camp settings, encourage parents to marry their daughters earlier to protect them from sexual violence before marriage despite international law that defines any girl under the age of 18 as a child and this form of marriage as sexually exploitative [26, 27, 32]. Similarly, parents in Syria often stated that rape or extramarital sex would disgrace their family and prevent future marriage, which led them to marry their daughters at a young age [27, 32]. A review of registered marriages in 2013 showed that one in every four Syrian refugee brides in Jordan was under the age of 18 [32]. In the same report, findings indicated that marriages are increasingly organized informally, which fosters the risk that girls are subsequently abandoned and divorced, and are unlikely to re-marry. Interviews with key informants as part of this research suggest that these short marriages are sometimes deemed “pleasure marriages” and informants speculated that the age gap between the Jordanian groom and Syrian bride is widening. Researchers suggested that this large age gap in marriages generally increases the power imbalance and can reduce young women’s autonomy, specifically her ability to refuse sex with her husband [32].

### Sexual exploitation of combatants

The second classification, sexual exploitation of combatants, was discussed in one-third of the studies [28, 30, 33, 34, 37, 35, 36, 38–44]. A majority of the study populations were former child soldiers [28, 36, 37, 39–43]. Rates of sexual abuse of female combatants ranged from 22–36% and males 5–57% [36–40]. Research indicated that girls were seen as replaceable and could be abducted in any number at anytime [28]. Study participants reported that it was common for girls to be purposely injured or threatened with guns to prevent escape [44].

All ten of the studies with evidence of sexual exploitation of combatants indicated that regardless of whether there was a forced marriage, a majority of former combatants were sexually abused [28, 30, 33, 35–41]. Denov’s in-depth interviews with victims recounted that former female combatants in Sierra Leone suggested that sexual violence was more debilitating than the violence inflicted during combat [42]. Three of the qualitative studies found evidence that combatant, who were also victims of sexual violence suffer alienation or stigmatization after their release [30, 41, 42].

### Sexual slavery

In the broadest category, sexual slavery, there were 10 studies that described perpetration of sex slavery outside of informal marriages, formal marriages and/or combatant conscription [20–23, 25, 30, 31, 45–47]. The evidence indicated that between 2–32% of individuals in conflict were victims of sexual slavery [20–22, 25, 45, 46]. Several studies reported prevalence for males and females separately, but the findings comparing the differences were inconsistent [21, 22].

In the Democratic Republic of Congo (DRC), Afghanistan and Côte d’Ivoire, qualitative evidence has been collected on women’s and girls’ accounts of abduction as sex slaves [30, 31, 47]. Analyses of interviews in Côte d’Ivoire suggest that women’s low status in society put them at greater risk [30]. Multiple studies indicated that victory in war often preceded incidents of abduction and rape as a form of celebration [30, 31]. The literature theorises that females had little to no autonomy and cites the use of the word ‘property’ as a reoccurring theme in survivors’ accounts of their period of abduction.

Considering the long history and far-reaching affect of conflicts, it is notable that there is an evidence base of only 29 studies, some notable methodological weaknesses and a range of incompatible indicator definitions. Despite the gaps in the evidence this review highlights that there is a convincing and growing body of evidence indicating that sexual exploitation and sex trafficking occur in conflict-affected settings and due to conflict-exacerbated risks. There are many questions left unanswered by this review pertaining to the prevalence and contributing factors that are critical to informing practice and policy, to be discussed in greater detail.

## Limitations of this review

The search strategy and quality assessment of this review were both conducted by a single reviewer. The reviewer followed strict inclusion criteria and the CASP quality assessment guideline to minimise bias. Additionally, due to the language limitations of the reviewers this review does not include studies or reports in languages other than English and there was not any grey literature searching in languages other than English.

## Discussion

Our findings confirm that the growing international attention to sex trafficking and sexual exploitation of individuals in conflict-affected settings is well-warranted [11]. This review indicates that women and girls, and men and boys, in situations of humanitarian crisis are subjected to extreme forms of sexual exploitation. Findings further highlight that, although men and boys may be victims of sexual abuses, women and girls are the most likely victims of targeted sexual violations in crises. The physical, sexual and mental health consequences of sexual abuses, especially among children, have been well-documented, particularly the psychological aftermath of sexual abuse, which very commonly includes post-traumatic stress disorder (PTSD), depression and suicidal ideation [48]. Even when the motives may be perceived as ‘protective’, such as in cases such as forced or early marriage of refugee girls, the health consequences are nonetheless likely to be difficult and enduring [49].

Additionally, this review demonstrates the paucity of evidence on sexual exploitation in conflict-affected settings and the particular weaknesses of current research to identify the prevalence of sex trafficking and similar sexual exploitation. It also highlights poor definitional clarity of trafficking-related terminology that is causing weak identification and measurement of exploitation, as well as disjointed programming in response to the problem. The challenges and gaps in this body of evidence suggest the need for more rigorous methods to measure and synthesize data. As a start towards better methodological approaches, this review has created an inventory of the divergences and overlaps in exploitation terminology that poses substantial challenges to data collection and analyses.

This review was limited in its attempt to measure prevalence and in the generalizations that can be concluded from such diverse and low quality studies. The quality of these studies, as described by the quality assessment, inhibits confidence in the findings in part due to the challenges associated with this type of sensitive research and in part because of the methodological weaknesses in defining the acts of sexual exploitation and limited capacity for large and generalizable samples.

Actions to address sex trafficking in conflict are clearly urgent. Recent reports by various humanitarian aid organizations and other reviews advise that international leaders initiate policy on human trafficking among refugees. In conflict settings, services are needed to assist individuals who are trafficking survivors and strategies must be developed to help prevent individuals from falling prey to those who will take advantage of the desperation of displaced populations [11, 50, 51]. Recommendations being made by humanitarian agencies and recently published reports are that counter-trafficking efforts need to be implemented immediately, if not before, the onset of conflict as a life saving precaution [11, 50, 51]. Increasingly, studies among refugees are describing the various ways that crises exacerbate individual vulnerabilities to various forms of exploitation and are calling for stronger evidence to inform policies and intervention approaches. For example, humanitarian aid workers require better information and training to be able to recognize and respond to sexual exploitation and trafficking among IDPs, in refugee camps and detention centers and among displaced populations in urban settings. Special measures will need to be put in place to help women and girls, in particular, and to support their families to prevent different forms of exploitation.

As noted, the robustness of future research, especially prevalence studies, will depend on deciphering the terminology and measurement tools around sex trafficking. Similarly, programme and policy responses will benefit from a deeper understanding of similarities and distinctions between the various forms of sexual exploitation. While interventions should aim to integrate responses to different forms of sex trafficking, such as early/forced marriage, sexual exploitation of combatants and sexual slavery, there should also be a clear understanding of the differing health, social and criminal justice needs required by survivors.

As international attention has recently turned towards refugees and the many conflict-affected settings, the time is right to focus squarely on human trafficking in these contexts. “We recognise that these crimes occur in situations where people are at their most vulnerable.” Developing more evidence-informed strategies to support trafficking survivors and prevent future victims is what is urgently needed.

## Conclusions

“Human trafficking and sexual exploitation occur in conflict-affected settings globally. Currently the evidence available indicates this is primarily occurring as early or forced marriage, forced combatant sexual exploitation, and sexual slavery. The studies in this review highlight the extraordinary vulnerability of women and girls to these extreme abuses and the severe health outcomes from exposure to this type of violence. It is critical and long overdue that humanitarian response should include both primary prevention and rehabilitation interventions for these forms of exploitation in the package of life-saving services delivered in conflict-affected settings. Simultaneously, this review suggests the need to clarify terminology around sex trafficking in conflict to foster a more cohesive future evidence-base, and in particular, robust prevalence figures from conflict-affected and displaced populations.

## Abbreviations

CASP: Critical appraisal skills program
CI: Confidence interval
DRC: Democratic Republic of Congo
FARDC: Forces Armées de la République Démocratique du Congo
GBV: Gender-based violence
LRA: Lord’s resistance army
LSHTM: London school of hygiene and tropical medicine
NGO: Non-government organizations
NYAM: New York academy of medicine
PTSD: Post-traumatic stress disorder
RHL: Reproductive health library
RUF: Revolutionary United Front
STD: Sexually transmitted disease
SWGA: Studies on women and gender abstracts
UN: United Nations
UNHCR: United Nations’ office of the high commissioner for human rights
UNODC: United Nations office on drugs and crime
WHO: World Health Organization
